# Application of a handheld Pressure Application Measurement device for the characterisation of mechanical nociceptive thresholds in intact pig tails

**DOI:** 10.1016/j.physbeh.2016.07.006

**Published:** 2016-10-15

**Authors:** Pierpaolo Di Giminiani, Dale A. Sandercock, Emma M. Malcolm, Matthew C. Leach, Mette S. Herskin, Sandra A. Edwards

**Affiliations:** a School of Agriculture, Food and Rural Development, Newcastle University, Newcastle upon Tyne NE1 7RU, United Kingdom; b Animal and Veterinary Science Research Group, Scotland's Rural College (SRUC), West Mains Road, Edinburgh EH16 4SA, United Kingdom; c Aarhus University, Department of Animal Science, AU-FOULUM, Tjele, Denmark

**Keywords:** Mechanical nociceptive thresholds, Pressure algometry, Pig

## Abstract

The assessment of nociceptive thresholds is employed in animals and humans to evaluate changes in sensitivity potentially arising from tissue damage. Its application on the intact pig tail might represent a suitable method to assess changes in nociceptive thresholds arising from tail injury, such as tail docking or tail biting. The Pressure Application Measurement (PAM) device is used here for the first time on the tail of pigs to determine the reliability of the methods and to provide novel data on mechanical nociceptive thresholds (MNT) associated with four different age groups (9, 17, 24 and 32 weeks) and with proximity of the target region to the body of the animal. We recorded an overall acceptable level of intra-individual reliability, with mean values of CV ranging between 30.1 and 32.6%. Across all age groups, the first single measurement of MNT recorded at region 1 (proximal) was significantly higher (*P* < 0.05) than the following two. This was not observed at tail regions 2 and 3 (more distal). Age had a significant effect (*P* < 0.05) on the mean thresholds of nociception with increasing age corresponding to higher thresholds. Furthermore, a significant effect of proximity of tail region to the body was observed (*P* < 0.05), with MNT being higher in the proximal tail region in pigs of 9, 17 and 24 weeks of age. There was also a significant positive correlation (*P* < 0.05) between mechanical nociceptive thresholds and age/body size of the animals.

To the best of our knowledge, no other investigation of tail nociceptive thresholds has been performed with the PAM device or alternative methods to obtain mechanical nociceptive thresholds in intact tails of pigs of different age/body size. The reliability of the data obtained with the PAM device support its use in the measurement of mechanical nociceptive threshold in pig tails. This methodological approach is possibly suitable for assessing changes in tail stump MNTs after tail injury caused by tail docking and biting.

## Highlights

• Mechanical nociceptive thresholds were quantified for the first time in pig tails.
• The PAM device allowed determining anatomical and age-specific thresholds in pigs.
• A platform for the assessment of painful conditions in pigs is proposed.

## 1 Introduction

The assessment of nociceptive thresholds is a recognized approach in the characterisation of sensitivity changes evoked by tissue damage [31]. In particular, the inflammatory responses associated with tissue damage may induce hyperalgesia (i.e. the increase in sensitivity to pain) or allodynia (i.e. the perception of pain from normally innocuous stimuli) [28]. Both these conditions can be quantified experimentally through sensory threshold testing comparing threshold values before and after the inflammatory event [2], [48], [55].

Despite the extensive use of nociceptive tests for quantification of conditions characterised by increased sensitivity in humans and rodents [31], its application has only recently been extended to include larger animal species (e.g. dogs, cattle, horses, sheep, poultry) [14], [23], [25], [44], [45], [49], [50]. Livestock, in particular, have received increasing attention due to their exposure to tissues damage and consequent inflammatory responses caused by husbandry procedures (e.g. castration, dehorning) and diseases (e.g. lameness) [8], [22], [32], [33], [64]. In pigs, the assessment of mechanical thresholds of nociception has been increasingly applied in the last decade, particularly in studies of lameness in sows and to evaluate the efficacy of analgesic protocols [38], [40], [43], [59]. While the mechanical nociceptive profiling of limbs in pigs is benefitting from an increasing body of research, little is known about thresholds of mechanical nociception in other anatomical regions. A few studies have provided novel data on the mechanical thresholds recorded in the flank of juvenile pigs [11], in the lower back of neonatal pigs [5], or at the tail root of juvenile [12], [54] and adult animals [39]. Increasing the knowledge on the variability of thresholds associated with specific features of the anatomical regions being investigated (e.g. tissue thickness, innervation) as well as the inclusion of factors such as age and body size would facilitate the use of algometry in the assessment of perturbations evoked by tissue damage (e.g. tail-docking, tail biting, castration) and in the development of porcine models of clinical pain research. Recent reports suggest that a greater level of repeatability may be obtained using a cuff-algometer (i.e. pneumatic actuators) applied to the limbs of adult sows [39] compared with handheld devices, which can be subject to some operator bias when applied incorrectly [46]. Despite this concern, we have previously reported some of the practical benefits of using a handheld Pressure Application Measurement device (PAM), applied to the hind limbs/flank of juvenile pigs, by controlling the rate of force delivered with the aid of visual feedback, hence reducing possible variability in application by the operator [11]. The PAM was originally designed for the application of a quantifiable, gradually increasing squeeze force for direct stimulation of the joint of rats [3] to allow measurement of mechanical nociceptive thresholds in experimental joint hypersensitivity models.

The aim of this study was to characterise mechanical nociceptive thresholds, with the use of a PAM device, in the intact tail of pigs at four different ages focussing on the following elements for analysis: (1) the degree of repeatability of nociceptive thresholds following the delivery of repeated stimuli to specific tail regions; (2) the influence of the stimulated tail region on thresholds of nociception recorded at sites varying in proximity to the body of the pig; and (3) the effect of age/body size on the mechanical nociceptive thresholds. This work was part of a research project aiming to provide knowledge for the development of protocols to assess acute and chronic pain associated with tail docking and tail biting in pigs.

## 2 Materials and methods

### 2.1 Animals and housing

All animal procedures were carried out under Home Office Licence (PPL 70/7919) and approved by the Animal Welfare Ethical Review Board (AWERB) of Newcastle University. A total of 123 female pigs, *Sus scrofa domesticus* (Landrace/Large White × synthetic sire line, Hermitage Seaborough Ltd., North Tawton, UK) from the resident herd at Cockle Park Farm, Newcastle University, were used in this study. The initial population of experimental animals comprised pigs of two distinct ages: (1) age of 9 weeks and a body weight of 22.3 ± 3.1 kg (*n* = 41); (2) age of 17 weeks and a body weight of 58.5 ± 8.4 kg (*n* = 82). The animals were originally selected for large investigation on changes in MNTs arising from tail resection, therefore only a sub-sample retained their tails until the age of 24 weeks and a body weight of 114.2 ± 12.9 kg (*n* = 16), selected from the 17 week-old population; and 32 weeks and a body weight of 152.3 ± 13.4 kg (*n* = 6), selected from the 9 week-old population. For the purpose of the research, all animals retained intact tails and were ear-tagged for identification within the first week of their life. Animals with tail damage were not selected and the appearance of any signs of tail and body injuries at any time previous to the test determined their exclusion. From selection at the time of farrowing until 1 week prior to the beginning of the study, the pigs were maintained under standard commercial conditions, with ad libitum access to feed and water. Due to the different experimental approach required (see below), the 9 week-old pigs received 60% of their normal daily rations in the home pen, with the remaining 40% provided as part of the habituation and test, starting 1 week prior to testing,. Throughout the experiment the temperature in the home pen was maintained at 19.0 °C (range: 18.0–20.0 °C). The experiment took place between December 2014 and October 2015.

### 2.2 Experimental design

The variability of thresholds of mechanical nociception was tested within the tail by obtaining measurements from three distinct regions (Region 1: proximal; Region 2: intermediate; Region 3: distal). Each region corresponded to approx. 1/3 of the entire tail length. All stimuli were delivered in triplicates at each tail region in rotation, so that two consecutive stimuli were never applied to the same tail region. This order guaranteed a minimum of 30 s intervals between each consecutive stimulus, with a total of approximately 2 min separating single stimuli to the same tail region. In order to examine variability of thresholds of nociception due to age/body size, data were collected in four age groups (9, 17, 24 and 32 weeks of age).

### 2.3 Experimental set-up

**Fig. 1:**
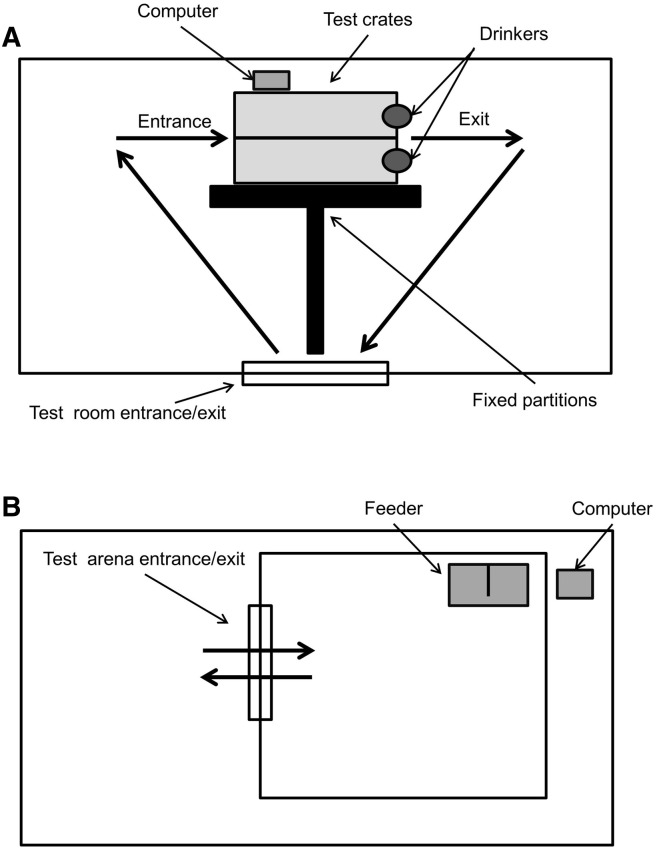
Outline of test set-ups. Set-up of the test room containing the test crates (A); test-arena (B). (1.5 column fitting image).

Pigs belonging to the 17, 24 and 32 week age groups were tested inside a custom-made crate situated in a test room adjacent to the home pens. The crate consisted of steel mesh panels as sides (1.8 m × 1 m) with a front and a rear gate, to allow the pigs to consistently enter at one end and leave from the other (Fig. 1). Through previous pilot studies, the size of the crate was determined as sufficient to provide confinement of the animals and prevent them from turning around. The crate incorporated a drinker fixed to the front gate, which contained a 5% sucrose solution. The concentration of sucrose solution was chosen because it is below that which can induce oro-gustatory analgesia in pigs [7]. Pigs of 9 weeks of age appeared to be too stressed by confinement in a crate, therefore testing of these animals occurred in a 3 × 3 m arena located in a room adjacent to the home pens (Fig. 1). The walls of the test arena consisted of 1 m high PVC boards, a height sufficient for the experimenter to reach the tail of the animals without being present in the arena. The arena consisted of a barren environment (concrete floor) with a feeder provided to the animals. For both situations, a computer was placed adjacent to the test area in a location that allowed the experimenter to observe the screen while concurrently applying the stimuli.

### 2.4 Nociceptive threshold testing device and equipment

Mechanical nociceptive thresholds of the tail were assessed using a handheld digital PAM device (Ugo Basile, Varese, Italy). The device consisted of a force-transducer assembly, which was originally designed to assess joint hypersensitivity in rodents [3], connected to an electronic unit recording the force applied by the operator. The transducer was equipped with a blunt-tipped acrylic probe with a diameter of 2 mm. Wearing the transducer on the thumb, the operator applied mechanical force at a rate of 120 g force (gF)/s. Calibration of the instrument was made by the manufacturer. The PAM device provides an output of the measurement in the unit of force (gF in this case), which does not take into account the pressure exerted by the instrument. Pressure is the results of force applied perpendicular to the surface per unit area over which it is distributed, in this case, 3.14 mm^2^. Force application was monitored on a laptop computer screen through dedicated software, which allowed the operator to match the actual rate of force application against the pre-set visualized value, to increase the stability of the stimulation. The maximum force was set at 1500 gF (14.7 N). This cut-off value was recommended by the manufacturer of the PAM device in order to guarantee the best level of stability of the force transducer and minimise the risk of tissue damage [53]. Stimulation was interrupted when a behavioural response occurred or when the cut-off force of 1500 gF was reached. The PAM device was operated by one investigator (PDG) throughout the study. Blinding was not possible due to the constraints of the study.

### 2.5 Habituation

Four days of habituation preceded the day of testing. During habituation, the pigs were allowed to walk freely in random pairs of pen-mates to the test setup (i.e. arena or crate) once daily. The pairs of pigs were confined for 7 min on the first day and the confinement period was increased gradually by 2 min per day, reaching a maximum of 13 min on day four. During habituation two observers followed the animals as they walked from the home pen to the test room and back and they were constantly present during the time of confinement. For the entire duration of the habituation period, 9 week-old pigs were provided with 60% of the daily feed ration through the feeder placed inside the test arena. Older animals assessed in the test crates were provided with sucrose solution via the drinkers.

### 2.6 Testing protocol

**Fig. 2:**
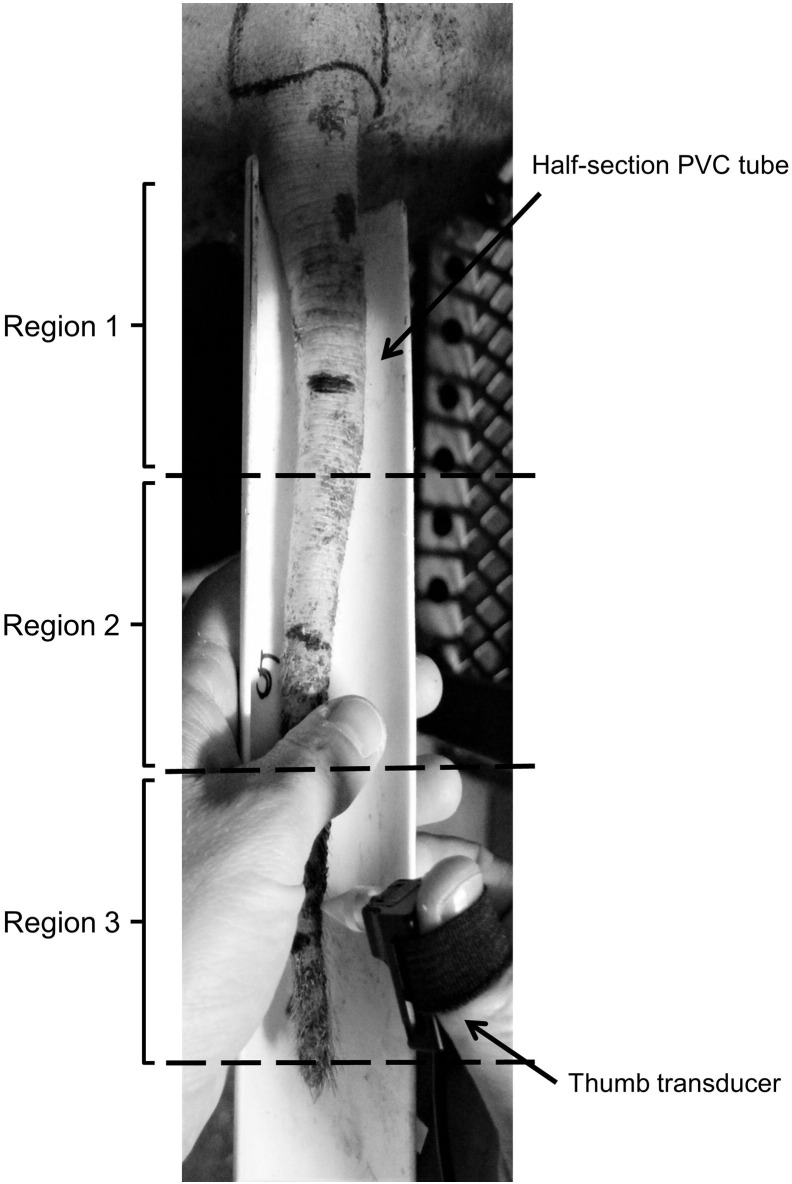
Image of the pressure application measurement (PAM) device applied to three distinct tail regions. (1 column fitting image).

Each pig was tested once at any given age, with one test session consisting of a total of nine mechanical stimuli applied in triplicate to each of three tail regions (Fig. 2). At the time of testing, two randomly-selected experimental pigs from the 9 week-old batch were allowed to walk freely from their home pen into the test arena. Testing began as soon as the animals started feeding. Similarly, older pigs were also allowed to walk freely from their home pen to the test crates in pairs with the test commencing immediately after both animals were fully confined in the crates and started drinking the sucrose solution. At the time of mechanical threshold testing, the tail was placed inside a half-section of PVC tube (length: 20 cm; diameter: 4 cm) (Fig. 2) by the operator, who gently applied downward force on the dorsal surface of the tail with one hand to allow the tail to extend fully inside the tube. This permitted maintaining the tail fully extended for several seconds, a time sufficient to deliver the mechanical stimuli to the dorsal surface. In case of the tail curling, the same operation was repeated as required. Two observable withdrawal responses elicited by the mechanical challenge were (1) a tail flick, defined as a rapid horizontal movement of the tail, and (2) a tail clamp, which consisted of a downward pressure applied by the tail to the tube and clearly detectable by the operator. These responses were previously described by Di Giminiani et al. [12]. In the absence of a response by the animal within the maximum cut-off value of 1500 gF, the measurement was recorded as 1500 gF but was excluded from the analysis. Once nine measurements per pig were obtained, the test session was concluded and the animals were released from the test confinement and returned to their home pen. A test session, comprising the time required for the pigs to reach the test room and return to their home pens, lasted on average 20 min.

### 2.7 Statistical analysis

In order to establish whether age and tail region influenced the degree of variability in thresholds, the coefficient of variation (CV) (i.e. the ratio of the standard deviation to the mean based on a minimum of two individual measurements), was calculated for each animal at each age group and tail region and it was compared through a two-way repeated measures ANOVA with age and tail region as fixed factors. Test-retest reliability within each session was evaluated with intra-class correlation coefficients (ICC) and their 95% confidence intervals for each tail region [63] and was included in the analysis as an indicator of within-session consistency of responses to individual mechanical stimuli. An ICC > 0.75 was considered to indicate excellent reliability, 0.40 to 0.75 fair-to-good reliability, and < 0.40 poor reliability [1]. Within-session effects of the repetition of stimuli were evaluated with an ANOVA on repeated measures for each tail region separately. The effects of age and tail region were evaluated with a two-way ANOVA on repeated measures with age and tail region as fixed factors. Finally, Pearson correlation coefficients were used to evaluate the relationship between pig body weight and mechanical nociceptive thresholds.

Results are presented as means ± SEM with significance at *P* < 0.05. All data were analysed using SPSS version 22.0 for Windows (SPSS Inc., Chicago, IL, USA).

## 3 Results

Out of a total of 1314 applied single stimuli, only 37 (2.8%) did not induce a clear and distinguishable withdrawal response. These were not associated with any specific tail region or animal age. The degree of within-session consistency varied substantially for each tail region and at each age. Values of ICC based on averages comprising the three tail regions were fair for the age groups 9, 17 and 24 (0.41, 0.46 and 0.41 respectively), while the lowest level of consistency was recorded in the 32 week-old pigs, with a poor overall ICC score (0.33) (Table 1).

Overall, despite the observed variation in consistency, within-session variability of MNTs did not differ significantly for different ages or tail regions, as indicated by the CV of individual animals (Table 2). Overall, the mean values of intra-individual coefficients of variation ranged between 32.6 and 30.1% across age groups and 32.8 and 30.9% across tail regions. On 11 occasions, the CV could not be calculated as only one successful measurement was obtained out of the three per region within an individual test session.

**Fig. 3:**
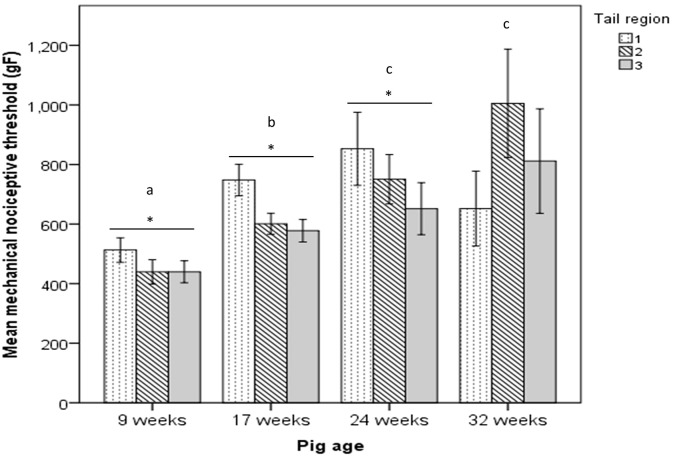
Mean mechanical nociceptive thresholds in intact pig tails. Mean mechanical nociceptive thresholds (gF) recorded at three tail regions (Region 1: proximal; Region 2: intermediate; Region 3: distal) and across four different ages (8, 16, 24 and 32 weeks of age). Different letters denote significant difference (*P* < 0.05) in thresholds between age groups. Asterisks indicate significant difference between tail region 1 and the following two regions within each age group (*P* < 0.05). (1.5 column fitting image).

For mechanical nociceptive thresholds (MNTs) there was a significant difference (*P* < 0.05) in single measurements recorded at region 1 (proximal) that was consistent across all age groups. Mean MNTs recorded as the first measurement (754.7 ± 29.9) were significantly higher (*P* < 0.05) than the mean of measurement of the second (674.7 ± 25.7) and third (656.4 ± 24.6) stimulus applications. There was no test-re-test effect within session observed in tail regions 2 and 3. A significant effect of proximity of tail region to the body was observed. Mean MNTs of 9, 17 and 24 week-old pigs were significantly higher in region 1 compared to region 2 and 3 (704.5 ± 22.5; 597.0 ± 16.0; and 556.3 ± 16.6 for Regions 1, 2 and 3 respectively; *P* < 0.05 for region 1 vs. 2 and 3; *P* > 0.05 for Region 2 vs. 3). In contrast, the same difference across tail regions was not observed in pigs tested at 32 weeks of age with mean thresholds of 652.1 ± 62.8 (region 1); 1005.1 ± 91.0 (region 2); 811.4 ± 87.8 (region 3) (*P* > 0.05 for all pairwise comparisons) (Fig. 3).

**Fig. 4:**
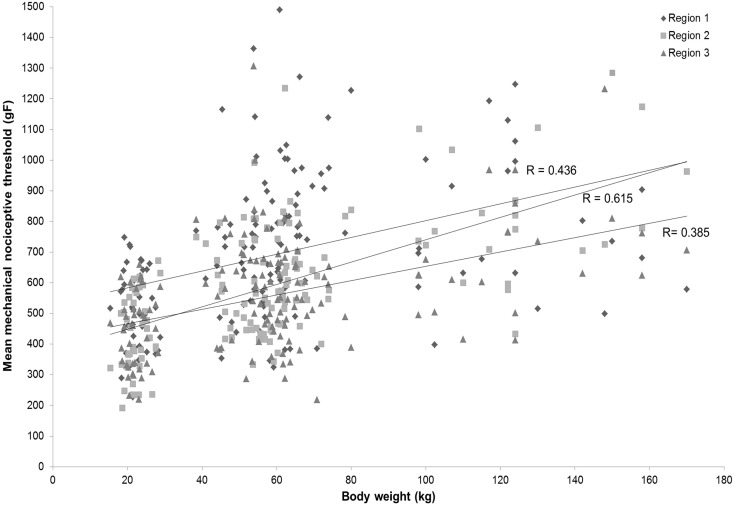
Correlation between mechanical nociceptive thresholds and age. A positive significant correlation between pig body weight (kg) and mechanical nociceptive thresholds (gF) was present within each tail region (Region 1: *r* = 0.385; Region 2: *r* = 0.615; Region 3: *r* = 0.436; *P* < 0.0001). (1.5 column fitting image).

Age had a significant effect (*P* < 0.05) on the mean thresholds of nociception with increasing age corresponding to higher thresholds, regardless of the specific tail region analysed (9 weeks: 464.0 ± 18.9; 17 weeks: 642.1 ± 13.4; 24 weeks: 751.6 ± 30.3; 32 weeks: 822.9 ± 49.4). All group ages differed significantly from each other (*P* < 0.05 for all pairwise comparisons), with the exception of 24 and 32 week-old pigs, which exhibited similar mean thresholds of nociception. A highly significant positive correlation (*P* < 0.0001) between pig body weight and mechanical nociceptive thresholds was observed within each tail region (Region 1: *r* = 0.385; Region 2: *r* = 0.615; Region 3: *r* = 0.436) (Fig. 4).

## 4 Discussion

This study demonstrated the feasibility of the assessment of mechanical nociceptive thresholds in the tails of pigs using a handheld PAM device. It allowed for the characterisation of MNTs and associated levels of variability and consistency of measurement in the pig tail in animals of different ages/body size and at specific regions along the tail. The results indicate that higher MNTs are associated with increasing age/body size in pigs. Furthermore, despite the relatively short distance between regions along the tail, it appears that thresholds obtained in more distal part of the tail were lower in comparison with more proximal tail regions, demonstrating that variations in MNTs exist even within small or discrete anatomical regions or sites.

To our knowledge, this is the first report on the measurement of MNTs in a longitudinal study on pigs with intact tails. Previous investigations have been carried out to quantify MNTs at the tail root or over more distal tail regions, but comprising exclusively pigs with docked tails [12], [39], [54]. In the present study, a level of responsiveness to individual stimuli of 97.2% was observed. This compares well with previous reports of 91.2% responsiveness to individual stimuli by Nalon et al. [39] when measuring MNTs on the ventral surface of docked tails in adult sows. The degree of variability observed in MNTs was approximately 30% CV across all age groups, which was consistent with values reported by Nalon et al. [39]: 38.9% in measurements obtained at the ventral aspect of the tail and 28.1% for the limbs. Several factors could contribute to the differences in variability reported across studies: anatomical differences, characteristic of the devices, experimental set-ups and protocols. Lower levels of variability in MNTs recorded for the tail region in the current study may have been the result of habituation of the pigs to the experimental procedure. Recently, Raundal et al. [46] reported that variability in MNTs could be reduced by habituating cows for few days before the test. Furthermore, familiarity with the experimental protocol has been suggested to improve the stability of the measurements in young pigs [26]. Habituation of the experimental animals should therefore be a fundamental element of the procedure. Where unfeasible due to time constraint, it should be estimated how the unfamiliarity of the animals could affect the measurements.

The characteristics of probes used to deliver mechanical nociceptive stimuli may be associated with different degrees of variability. In the current study, the choice of the probe was based on the induction of clear behavioural withdrawal responses without any signs of superficial tissue damage even following several applications. In horses, variability in mechanical nociceptive thresholds has been estimated for probes of different sizes, suggesting that smaller areas of force application increased the reliability of the measurements [60]. The reliability of the measurements may also be determined by the stability of the mechanical stimuli, which directly relates to the operator and the specifications of the devices delivering the challenge. Irregularities in the rate of force increase have been indicated as one of the potential causes of variability in mechanical nociceptive thresholds recorded in piglets [26], as well as in horses [21]. In recent years, handheld devices have improved to provide the possibility of controlling the rate of force application, in an attempt to reduce fluctuations and instability. Appropriate training of the operator benefits the stability of the ramped stimuli in humans [62]. In addition to training, handheld devices used in large animal research can be fitted with warning lights alerting the operator when the mechanical stimuli are not applied at a constant increasing force rate [20], [39]. The advantage of the PAM device is that it provides a visual feedback of the force applied by the operator against a pre-set rate on a computer monitor, thereby reducing deviations from the intended rate.

The observed differences in mechanical nociceptive thresholds recorded at three different tail regions may reflect different degrees of somatosensory innervation across the tail and/or site-specific morphological differences in organ/tissue composition (e.g. skin, subcutaneous fat and muscle thickness). Anatomical region-specific effects on mechanical thresholds have been previously described following measurements obtained in several landmarks within the back of horses [21], [37], the back and limbs of dogs [9] and the flank and limbs of pigs [11]. Nevertheless, this is the first report on variations in nociceptive threshold measures obtained within a limited anatomical area, such as the tail. In sheep, mechanical thresholds recorded on the hind legs differed between adjacent points [58]. Similar within-region variation has been reported in cows [45], and pigs [39], with different thresholds recorded between the lateral and the dorsal aspects of the same limbs. In humans, mechanical nociceptive thresholds differed when measured over bony prominences or muscles [52]. Similarly, inter-site differences have been reported at different depths of soft skin tissues in horses [34]. The pig tail, despite the more consistent underlying anatomical structures, appears to be characterised by a similar high degree of site specificity. In rats, exposing the distal portion of the tail to a thermal challenge induced more rapid responses (i.e. tail flick reflex) as opposed to a proximal area of stimulation [41]. The results of the current study may be explained in part by a variation in the somatosensory innervation of the skin. A proximal-distal gradient in densities of epidermal nerve fibres has previously been reported in humans with greater densities measured in the distal part of the leg [36]. Regional histological and immunological characterisation of tail innervation in the future may provide insight into possible somatosensory differences that may explain, in part, the proximal to distal differences observed in MNTs in pig tails. Although it is recognized in humans that the correlation between nociceptive thresholds and density of nerve fibres is not linear [29], [56], this has not been investigated in animals. The possibility of obtaining different thresholds within limited anatomical areas warrants a careful evaluation of the appropriate size of the stimulation area. Repeatedly applying stimuli to the same point of contact on the animal may induce spatial and/or temporal summation at the synaptic level [42], [57], causing a change in the baseline values of mechanical thresholds. To avoid this, the stimuli should be applied within a relatively small area with sufficient time intervals between them. Although inter-stimuli intervals of 1–2 min have been suggested to be sufficient to avoid temporal summation in pigs [11], [39], it is presently unclear what the optimal stimulation area is in larger animals, thus supporting the need for a more comprehensive understanding of the site-specific distribution of nociceptive thresholds in different anatomical regions.

The reported difference in MNTs associated with distinct animal ages in the present study may be due to age-specific anatomical characteristics (e.g. thickness of the skin, subcutaneous fat and muscles) as well as different sizes of nociceptor receptive fields and changing stages of maturation of the nociceptive system. Because of the positive correlation between age and body weight in pigs, both elements could contribute to the observed changes in nociceptive thresholds. Positive association between nociceptive thresholds and body weight has been reported in cats [13] and dogs [4]. Similar correlations between anthropometric variables (i.e. body mass index) and thresholds of nociception have been reported in humans [47]. Age-related changes in thresholds have been observed in rats [6], [27] and reports in humans indicate that thermal and mechanical thresholds increase with age [30], [51]. Greater pig body weights may be related to deeper thickness of soft tissues, which may consequently require the delivery of mechanical stimuli of greater force. Distinct values of mechanical nociceptive thresholds have been linked to the varying thickness of subcutaneous adipose tissues in humans [16]. Furthermore, the propagation of mechanical pressure is dependent on the muscle tissue involved in the stimulation [17], which may thus contribute to the observed difference in mechanical thresholds at the tail of pigs. Finally, these differences may be attributed to age-specific maturational changes in neural circuitry, which have been reported in rats [10] and in humans [18], [24]. Information from previous reports on mechanical nociceptive thresholds over a wide range of age/body size [11], [26], [39], [53], together with the current results, suggest that a positive correlation between age/body size and nociceptive thresholds exists in pigs.

In this study, the lack of a significant difference in mechanical nociceptive thresholds recorded between pigs of 24 and 32 weeks of age should be interpreted with caution. Although data from the oldest animals appear to conform to the positive correlation between age/body size and thresholds, they are influenced by a combination of low animal sample size and wide confidence intervals. Furthermore, it cannot be excluded that the observed differences in mechanical nociceptive thresholds become less detectable when the difference in body weight is of lower magnitude between animals of 24 and 32 weeks of age compared to the younger age groups. Future studies should focus on the temporal changes in nociceptive thresholds, which would facilitate the selection of appropriate ranges of animal ages as well as identify the most suitable characteristics of the instruments used (e.g. magnitude of application force).

Modified versions of the PAM device with increased force transducer cell loads and stimuli delivery systems have previously been used in studies on large farm species including pigs [11] and dairy cows [45]. In the present study the delivery of mechanical stimulation to the pig tail was achieved using the original thumb transducer [3]. Application of the stimuli using this approach, along with how the tail was manipulated, conferred some advantages by improving the stability of force application and removing the need for restraint of the tail by the operator. This contrasts with the methodological approach to the measurement of tail MNTs in sows in a recent study by Nalon et al. [39] where the tail was lifted up by the operator and restrained by hand during the application of the stimulus, which may affect the ability of the tail to reflexively react to the stimuli (e.g. diminished effector responses due to tail posture) and cause unwanted and confounding mechanical stimulation in areas not directly stimulated by the device.

One of the potential limitations of mechanical nociceptive methodologies could be reflected in the different amount of force required to evoke a response between the first and the following two stimuli delivered to the rail region proximal to the body. As highlighted by Le Bars et al. [31], the initial response to a mechanical challenge may be caused by a pure spinal reflex, without involvement of higher spinal and supraspinal structures, followed by enhanced responses possibly caused by the animal learning to recognise the stimulus. Nonetheless, this was only observed in one tail region and the stimuli were delivered at random across the three regions. An alternative explanation to the learning effect may be the nociceptive sensitisation induced by the greater force of the initial stimulation. The greater degree of force, associated with greater tissue strain and stress, provided by the first force application may have not only triggered cutaneous afferents [35], but also deeper tissue responses. In humans, involvement of deeper tissues has been suggested as the cause of summation [15]. The involvement of muscle primary sensory afferents has been reported as a key contributor to lowered nociceptive thresholds to noxious mechanical stimulation in rats [61].

A potential limitation in this study was the use of the two different test set-ups. All animals were intended to be habituated and tested in the crates. However, based on observations from pilot studies, pigs of 9 weeks of age did not readily accept confinement (i.e. high frequency of escape attempts) even following several days of habituation. Due to the increased fear responses observed in younger pigs [19], we opted instead for an open test arena. Although the relative degree of confinement experienced may have had an effect on the demeanour of the pigs, all the other experimental conditions (i.e. habituation, presence of companion animal, motivation through food/sucrose solution) were the same in the two test set-ups. Testing of pigs in an open arena may potentially cause less stress to animals of this age than testing in a crate. However, it has been previously demonstrated that familiarity with the experimental set-up markedly improves the performance of the animals in tests of nociception performed in a crate by reducing the effect of stress [12]. In contrast, habituation was not observed to affect mechanical nociceptive thresholds recorded in an open arena, which suggests that in this particular study, potentially stressful effects of testing in an open area may have been negligible. Future investigations on the influence of the degree of animal confinement on measures of nociception are recommended.

## 5 Conclusions

In conclusion, this study demonstrated the suitability of the use of the PAM device for measuring mechanical nociceptive thresholds in intact tails of pigs of different ages and body sizes. The efficacy in inducing clear withdrawal tail responses and the acceptable level of variability and consistency support the application of this handheld methodology for the assessment of changes in tail nociceptive thresholds potentially arising from tail docking and tail biting in pigs. Furthermore, the current results suggest that there is positive correlation between mechanical nociceptive thresholds and age/body size in a previously unexplored anatomical region, thus contributing to the growing body of information necessary to extend the application of nociceptive testing methodologies in pigs.

## Conflict of interest

No conflict of interest to report.

## Figures and Tables

**Table 1:** Intra-rater, within-session reliability of mechanical nociceptive threshold measurements at each tail region (ICC with 95% confidence intervals).

Age	Sample size	Region 1 (proximal)	Region 2 (mid)	Region 3 (distal)	Average
9 weeks	41	0.45 (0.05–0.69)	0.51 (0.15–0.73)	0.28 (− 0.28–0.61)	0.41
17 weeks	82	0.55 (0.35–0.70)	0.38 (0.09–0.58)	0.46 (0.21–0.63)	0.46
24 weeks	16	0.59 (0.07–0.85)	0.14 (− 0.99–0.67)	0.48 (− 0.20–0.80)	0.41
32 weeks	6	0.10 (− 2.82–0.86)	0.46 (− 1.29–0.92)	0.42 (− 1.46–0.91)	0.33

**Table 2:** Mean within-session variability of mechanical nociceptive thresholds for each age group and tail region. The intra-individual coefficient of variations (CVs) is based on three individual measurements per pig and presented as the mean and standard errors (SEM).

Pig age	Mean	Std. error	95% confidence interval	
			Lower bound	Upper bound
9 weeks	0.316	0.016	0.285	0.347
17 weeks	0.326	0.012	0.302	0.349
24 weeks	0.301	0.025	0.252	0.349
32 weeks	0.311	0.040	0.232	0.390

Tail region	Mean	Std. error	95% confidence interval	

Lower bound	Upper bound

1	0.328	0.022	0.284	0.371
2	0.303	0.022	0.259	0.347
3	0.309	0.022	0.266	0.353
